# Incorporation of a Dietary Omega 3 Fatty Acid Impairs Murine Macrophage Responses to *Mycobacterium tuberculosis*

**DOI:** 10.1371/journal.pone.0010878

**Published:** 2010-05-28

**Authors:** Diana L. Bonilla, Lan H. Ly, Yang-Yi Fan, Robert S. Chapkin, David N. McMurray

**Affiliations:** 1 Department of Microbial and Molecular Pathogenesis, College of Medicine, Texas A&M Health Science Center, College Station, Texas, United States of America; 2 Program in Integrative Nutrition & Complex Diseases, Texas A&M University, College Station, Texas, United States of America; 3 Faculty of Nutrition, Texas A&M University, College Station, Texas, United States of America; 4 Center for Environmental and Rural Health, Texas A&M University, College Station, Texas, United States of America; Statens Serum Institute, Denmark

## Abstract

**Background:**

Beside their health benefits, dietary omega 3 polyunsaturated fatty acids (n-3 PUFA) might impair host resistance to *Mycobacterium tuberculosis (Mtb)* by creating an immunosuppressive environment. We hypothesized that incorporation of n-3 PUFA suppresses activation of macrophage antimycobacterial responses and favors bacterial growth, in part, by modulating the IFNγ-mediated signaling pathway.

**Methodology/Principal Findings:**

Murine macrophage-like J774A.1 cells were incubated with bovine serum albumin (BSA)-conjugated docosahexaenoic acid (DHA; 22:6n-3) or BSA alone, activated with recombinant IFNγ, and infected with a virulent strain (H37Rv) of *M. tuberculosis*. The fatty acid composition of macrophage membranes was modified significantly by DHA treatment. DHA-treated macrophages were less effective in controlling intracellular mycobacteria and showed impaired oxidative metabolism and reduced phagolysosome maturation. Incorporation of DHA resulted in defective macrophage activation, as characterized by reduced production of pro-inflammatory cytokines (TNFα, IL-6 and MCP-1), and lower expression of co-stimulatory molecules (CD40 and CD86). DHA treatment impaired STAT1 phosphorylation and colocalization of the IFNγ receptor with lipid rafts, without affecting surface expression of IFNγ receptor.

**Conclusions/Significance:**

We conclude that DHA reduces the ability of J774A.1 cells to control *M. tuberculosis* in response to activation by IFNγ, by modulation of IFNγ receptor signaling and function, suggesting that n-3 PUFA-enriched diets may have a detrimental effect on host immunity to tuberculosis.

## Introduction

Tuberculosis (TB), a bacterial disease caused by *Mycobacterium tuberculosis (Mtb)*, continues as a public health problem [1]. Host resistance to this pathogen requires the development of an effective cellular immune response, mediated mainly by T cells and macrophages [1]. The activation of these cells can be modulated by nutritional components, such as carbohydrates, proteins and vitamins, impacting subsequently on the resistance to TB [2], [3], [4]. On the other hand, lipids can regulate the host response against *Mtb*. Fatty acids such as arachidonic acid increase bacterial killing in macrophages [5]; while cholesterol impairs immunity against *Mtb* [3]. Moreover, it was recently reported that eicosapentaenoic acid (EPA), an n-3 polyunsaturated fatty acid (n-3 PUFA), increased mycobacterial growth by reducing TNFα secretion in macrophages [6]. However, the role of distinct classes of long chain dietary fatty acids during TB infection has not been well investigated to date.

Dietary n-3 PUFA are found in cold water fish and fish oils and their effect on immune responses has been studied extensively. The anti-inflammatory properties of n-3 PUFA make them useful as a therapy for chronic inflammatory diseases, autoimmune disorders and cancer [7], [8], [9]. However, epidemiological studies have linked n-3 PUFA-enriched diets not only with a reduced incidence of inflammatory diseases but also with an increased incidence of TB [10]. We previously aerosol challenged transgenic fat-1 mice, which endogenously produce n-3 PUFA, and fish oil-fed guinea pigs with H37Rv *Mtb* and bacteriological and histological analysis revealed that n-3 enrichment enhances susceptibility to TB infection, as demonstrated by higher bacterial loads and less robust inflammatory responses in lungs [11],[12].

Macrophages are both host cells for *Mtb* and effector cells for host resistance, being responsible for mycobacterial killing. The effectiveness of the macrophage antimycobacterial activity depends on an appropriate level of cell activation [13],[14]. IFNγ activates macrophage maturation [15], inducing upregulation of proinflammatory cytokines and surface markers. However, n-3 PUFA have been found to impair IFNγ-induced activation [16] thereby reducing the ability to kill some pathogens [17] and to stimulate an acquired immune response [18]. Different processes important for killing activity are affected by DHA, including the respiratory burst [19], chemotaxis [20], antigen presentation [21], the expression of adhesion molecules [18] and major histocompatibility complex (MHC) [18],[22]. DHA-treated cells also produce less proinflammatory eicosanoids [23] and cytokines [24] which are important for a protective response against TB [25], [26]. Furthermore, lipids can influence phagolysosome maturation [5] and endosomal membrane lipid composition [27], which is critical for mycobacterial clearance

We demonstrate that IFNγ-treated macrophages cultured *in vitro* with DHA are more permissive to *Mtb* infection in association with several measurements of defective cell activation. The reduced capacity of DHA-treated, IFNγ-activated macrophages to control the infection was accompanied by defects on proinflammatory cytokine production, phagolysosome maturation, and oxidative burst. Furthermore, DHA significantly affected the early events of cell signaling in IFNγ-treated macrophages which may explain, in part, the negative effects of DHA on macrophage resistance to *Mtb*.

## Results

### DHA treatment alters lipid membrane composition

J774A.1 cells efficiently incorporate fatty acids provided in the culture medium into their cell membranes under the conditions used in this study. **Table 1** shows that DHA-treated cells significantly (p<0.05) increased the DHA content into their membrane phospholipids. The amount of DHA (22:6n-3) in DHA-treated cells was 18.2±0.5 mol%, compared to 2.15±0.50% in cells incubated with medium alone (Control). We and others have reported similar levels of DHA, exceeding 10%, on both total and phospholipid fractions in peritoneal macrophages and splenic T-cells from mice fed diets enriched with DHA [28], [29], [30], [31].

**Table 1:** Fatty acid composition in phospholipid fractions from fatty acid-treated J774A.1 cells.

	Treatments	
Fatty Acid	Control	DHA
**14:0 (myristic acid)**	3.55±0.15	3.76±0.13
**14:1 (myristoleic acid)**	2.16±0.37	2.09±0.26
**16:0 (palmitic acid)**	27.11±0.43	31.04±.032
**16:1n-7 (palmitoleic acid)**	8.91±0.23	6.40±0.01
**18:0 (stearic acid)**	10.44±0.28	9.46±0.20
**18:1n-9 (oleic acid)**	20.93±0.32	13.36±0.15
**18:1n-7 (vaccenic acid)**	10.15±0.32	6.91±0.10
**18:2n-6 (linoleic acid)**	5.56±0.79	2.69±0.06
**20:4n-6 arachidonic acid)**	5.74±0.59	5.01±0.06
**22:4n-6 (adrenic acid)**	0±0	0±0
**22:5n-6 (docosapentaenoic acid)**	0.84±0.02	0±0
**22:5n-3 (docosapentaenoic acid)**	1.49±0.39	1.07±0.00
**22:6n-3 (docosahexaenoic acid-DHA)**	2.15±0.50	18.24±0.52*

Cells were incubated with 50uM DHA for 24 hours and the lipid incorporation was assessed by gas chromatography, as described in the *Materials and Methods*. Results are expressed as mol per 100 mol of total fatty acids and only selected major fatty acids (>1mol%) are reported. Values represent mean ± SE (n = 3).

*Significantly different from Control (Cells treated with no fatty acids) in the same fraction, p<0.05. Abbreviations used: DHA, docosahexaenoic acid.

### DHA treatment impairs macrophage activation

**Figure 1:**
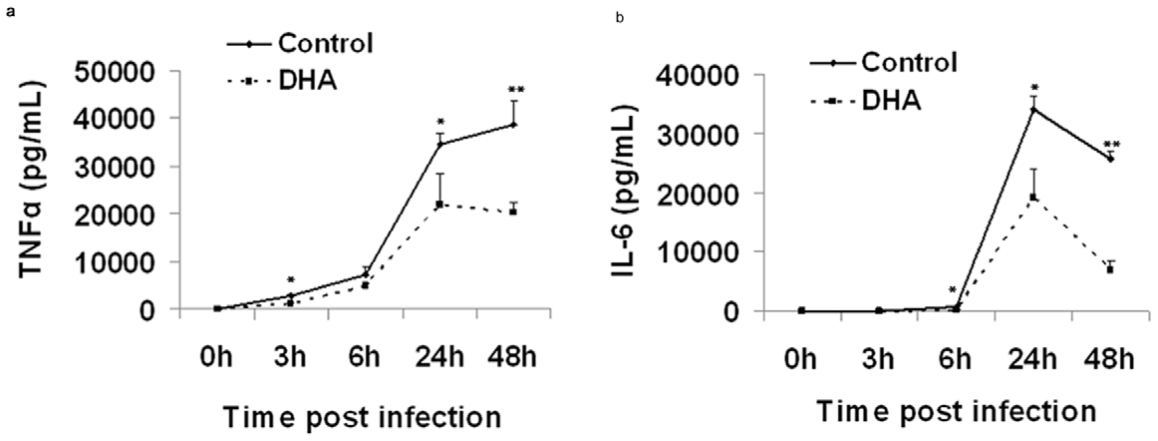
Suppression of TNFα and IL-6 by DHA in infected J774A.1 cells after IFNγ treatment. Macrophages were incubated with 50uM DHA for 24 h and infected with *M. tuberculosis* for 1 h. Culture supernatants were quantified for TNFα and IL-6, as described in the *Materials and Methods*. Cells were originally seeded at a density of 250,000 cells/well. **a.** Quantitative data represent the concentration of TNFα and **b.** IL-6 post infection (pictograms per milliliter; mean ± SEM; n = 9).* Indicates a significant effect of DHA within the same interval compared to Control, * p<0.05 ** p<0.01. **c.** Representative histogram of the reduced IL-6 levels (lower intensity of fluorescence) in DHA-treated cells, compared to Control at 6 h post infection. The analysis was restricted to the fluorescent beads coated with antibodies to IL-6. Data are representative of three independent experiments. Abbreviations used: DHA, docosahexaenoic acid.

In order to examine the effect of DHA incorporation on activation of infected J774A.1 cells, we measured the production of proinflammatory cytokines and expression of costimulatory molecules. IFNγ-induced activation of infected cells was impaired significantly by DHA. As seen in **Figure 1**, there was significant upregulation of TNFα between 24 h and 48 h and IL-6 at 24 h in infected cells. DHA significantly reduced TNFα protein levels at 3, 24 and 48 h in infected IFNγ-stimulated cells (**Fig. 1A**). For example at 24 h, DHA treatment resulted in a 37% reduction in TNFα production, compared to control. In panel B, DHA significantly reduced IL-6 levels at 6, 24 and 48 h (**Fig. 1B**). The percentage of DHA-induced reduction was 74% at 48h (p<0.01). We observed a similar suppressive effect of DHA after LPS treatment and on MCP-1 levels (Data not shown). The production of IL-12p70, IL-10 and IFNγ was not affected by DHA under our experimental conditions.

**Figure 2:**
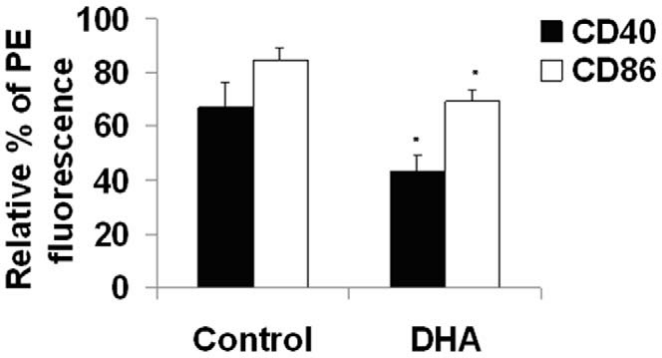
DHA reduces expression of CD40 and CD86 in infected J774A.1 cells primed with IFNγ. Macrophages were incubated with 50uM DHA for 24 hours and infected with *M. tuberculosis* for 1 h. Then cells were incubated with PE-conjugated antibodies to CD40 and CD86 and intensity of fluorescence was quantified by flow cytometry, as described in the *Materials and Methods*. Cells were seeded at a density of 500,000 cells/well. a. Quantitative data represent the relative percentage of PE fluorescence (mean ±SEM; n = 9) for CD40 and CD86. * Indicates a significant effect of DHA compared to Control, * p<0.05. b. Comparative histograms of CD40 and CD86 expression in fatty acid-treated cells in DHA-treated cells after 24 h, compared to other fatty acid treatments. Data are representative of three independent experiments. Abbreviations used: DHA, docosahexaenoic acid.

DHA significantly suppressed upregulation of CD40 and CD86 in infected cells primed with IFNγ. For example, **Figure 2** shows significant (p<0.05) differences in the CD86 percentage of PE fluorescence between DHA (69.3±4.5%) and control (84.6±4.6%), as well as 35% reduction in CD40 expression. No significant changes in CD14 expression were observed (data not shown). The observed differences in cytokine production and surface molecule expression were not explained by changes in cell viability, proliferation or adherence (data not shown). We confirmed that these effects were specific for DHA by comparing the same responses in cells treated with two control fatty acids, AA and PA. J774A.1 cells treated with AA or PA did not exhibit significant changes in TNFα production or CD40/CD86 expression (**Figure S1a**). As shown in **Figure S1b**, significant (p<0.05) differences in the CD86 percentage of PE fluorescence between DHA (69.3±4.5%) and other treatments (Control 84.6±4.6%, AA 89.6±2.3, PA 84.3±4.2).

### DHA reduces macrophage ability to control *M. tuberculosis* infection

**Figure 3:**
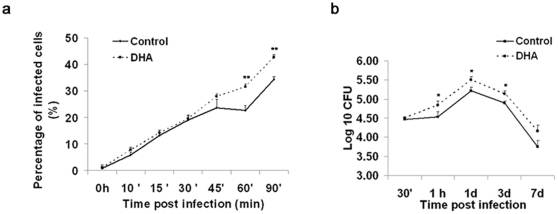
DHA reduces mycobacterial killing in IFNγ-stimulated J774A.1 macrophages. Cells were incubated with 50uM DHA for 24 h and infected with10 MOI GFP-expressing *M. tuberculosis* for 1 h. **a.** The relative percentage of *Mtb*-infected cells was quantified by flow cytometry, as described in the *Materials and Methods*. Quantitative data represent the percentage of infected cells (mean ±SEM; n = 9). DHA increases bacterial survival in infected J774A.1 cells. **b.** Bacterial counts were estimated at different time points by the CFU assay, as described in the *Materials and Methods*. Data show bacterial survival as Log10 CFU (mean ± SEM; n = 10). Cells were seeded at a density of 250,000 cells/well. * Indicates a significant effect of DHA compared to other treatments, * p<0.05, **p<0.01. Abbreviations used: DHA, docosahexaenoic acid.

In order to determine if DHA incorporation impaired the ability of IFNγ-treated J774 cells to control *Mtb*, we measured intracellular bacterial accumulation by flow cytometry and bacterial survival by the colony forming unit (CFU) assay. **Figure 3A** shows that the percentage of infected cells increased with time in all treatment groups. DHA-treated cells were more permissive to the infection as 31.6±1.4% of the cells were infected after 60 min, using MOI of 10; reaching 42.3±1.6% of infection over 90 min whereas infection in the control group only reached 22.4±0.9% and 33.2±0.4%, respectively (p<0.01). The differential bacterial accumulation was confirmed by fluorescence microscopy (Data not shown). Moreover, DHA facilitated bacterial persistence within macrophages. As shown in panel B, a greater number of viable mycobacteria were recovered from DHA-treated cells at all intervals (30 min, 1 h, 1, 3 and 7 d), although statistical significance was only achieved at 1 h, 1, and 3 d (p<0.05). J774A.1 cells treated with AA or PA did not exhibit significant changes in bacterial growth (**Figure S2a**).

### DHA impairs phagolysosome maturation

**Figure 4:**
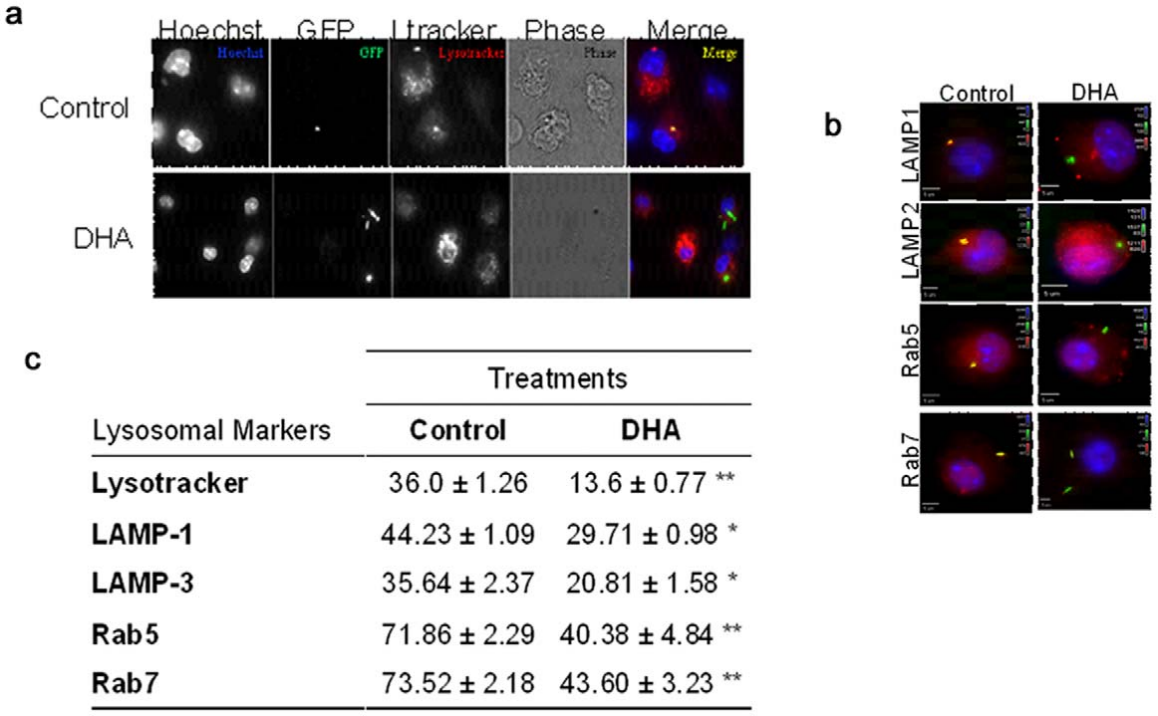
DHA reduces mycobacterial phagolysosome maturation in IFNγ-primed J774A.1 cells. Cells were incubated with 50uM DHA for 24 h and infected with20 MOI GFP-expressing *M. tuberculosis* for 1 h. Cells were seeded at a density of 500,000 cells/well. Phagolysosome maturation was defined based on acquisition of lysotracker by GFP-*Mtb*-containing phagosomes and visualized by fluorescent microscopy, as described in the *Materials and Methods*. a–b. Representative images of the reduced colocalization induced by DHA, compared to Control. Data representative of three independent experiments. c. Percentages of Lysotracker-, LAMP-1, LAMP3-, Rab5-, Rab7-positive mycobacterial phagosomes. Data represent the percentage of colocalization (mean ±SEM; n = 5). * Indicates a significant effect of DHA compared to mock control, *p<0.05, **p<0.01. Abbreviations used: DHA, docosahexaenoic acid; LAMP-1, lysosomal-associated membrane protein.

Since DHA impaired bacterial control in IFNγ-treated cells, we focused on cell processes which are enhanced by IFNγ and fundamental to the control of intracellular mycobacteria. The fusion of *Mtb*-containing phagosomes with compartments of the endocytic pathway plays an important role in antimycobacterial activity by releasing the lysosome content into the mycobacterial compartment. In order to determine the effect of DHA on phagosomal maturation, we assessed the incorporation of the acidotropic probe lysotracker into mycobacterial phagosomes. IFNγ treatment resulted in increased colocalization of *Mtb* and lysotracker (MOI 20) (Control 11.3±2.5% vs IFNγ 58.4±3.8%). We observed reduced lysotracker colocalization in IFNγ-primed cells treated with DHA (**Fig 4A**), showing that the activating effect of IFNγ was significantly reduced by DHA. DHA induced a 62.22% reduction in lysotracker-positive mycobacterial phagosomes over 1 h after infection (**Fig. 4C**; DHA 13.6±0.8% vs Control 36.0±1.3%, p<0.01). Additionally, we examined maturation of phagosomes by monitoring acquisition of early (Rab5) and late endosomal markers (LAMP1, LAMP-3 and Rab7). **Figure 4B** shows representative fluorescent images of the reduced recruitment of LAMP1, LAMP-3, Rab-5, and Rab-7 to mycobacterial phagosomes in DHA-treated cells. Panel C summarizes the percentage of *Mtb*-containing phagosomes colocalizing with lysosomal markers. We observed significantly reduced phagolysosome maturation in infected IFNγ-primed cells treated with DHA. DHA treatment resulted in significantly decreased presence of Lamp-1 (DHA 29.7±1.0% Vs Control 44.2±1.1%; p<0.05), Lamp-3 (DHA 20.8±1.6% Vs Control 35.6±2.4%) and Rab7 (DHA 29.1±1.6% Vs Control 49.0±1.1%; p<0.01) in mycobacterial phagosomes. We confirmed that DHA-induced alterations in mycobacterial infection and phagolysosomal maturation were specific for an n-3 PUFA. As show, in **Figure S2b**, no significant changes in either of these parameters were observed in J774A.1 cells treated with AA or PA.

### DHA suppresses the respiratory burst

**Figure 5:**
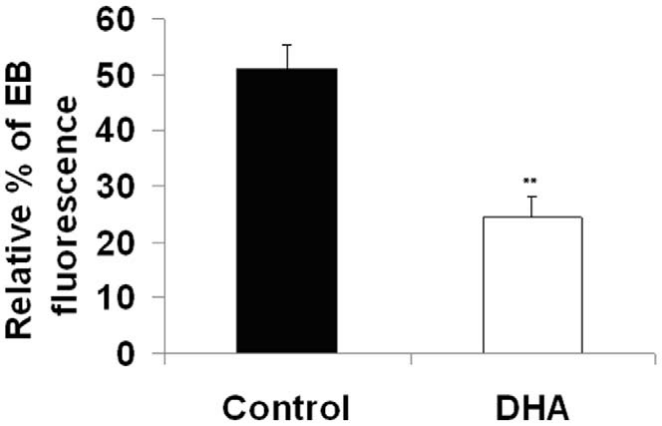
DHA impairs oxidative metabolism in infected J774A.1 cells under IFNγ treatment. Cells were incubated with 50uM DHA for 24 h and infected with 20 MOI of *Mtb*. Reactive oxygen intermediates were estimated by FACS, as described in the *Materials and Methods*. **a.** Quantitative data show the relative percentage of EB red fluorescence (mean ± SEM; n = 9). * Indicates a significant effect of DHA compared to Control, **p<0.01. Abbreviations used: DHA, docosahexaenoic acid; EB, ethydium bromide.

The generation of reactive intermediates derived from oxygen or nitrogen is necessary for resistance to TB. In order to determine the effect of DHA on oxidative metabolism, we assessed the ability of DHA-treated macrophages to generate ROS following infection. In the presence of DHA, IFNγ-treated *Mtb*-infected J774A.1 cells exhibited significantly (p<0.01) reduced oxidative activity (MOI 10). **Figure 5** shows that cells treated with DHA exhibited a significantly decreased percentage of EB fluorescence (DHA 24.4±3.7% vs Control 51.2±4.2%; p<0.01). The dye used, dyhydroethidium (DHE), is oxidized by ROS to ethidium bromide (EB), which emits red fluorescence. Background oxidation of DHE occurred due to macrophage metabolic activity (uninfected cells 11.4±4.7). The suppressive effect was also seen following LPS treatment (Data not shown). Overall, these data suggest that the increased bacterial loads found in DHA-treated cells might be explained by an impaired oxidative burst. We confirmed that these effects were specific for DHA by comparing the same readouts in J774A.1 cells treated with AA or PA. As shown in **Figure S3**, the only significant treatment effect was observed with DHA treatment.

### DHA downregulates cell signaling

**Figure 6:**
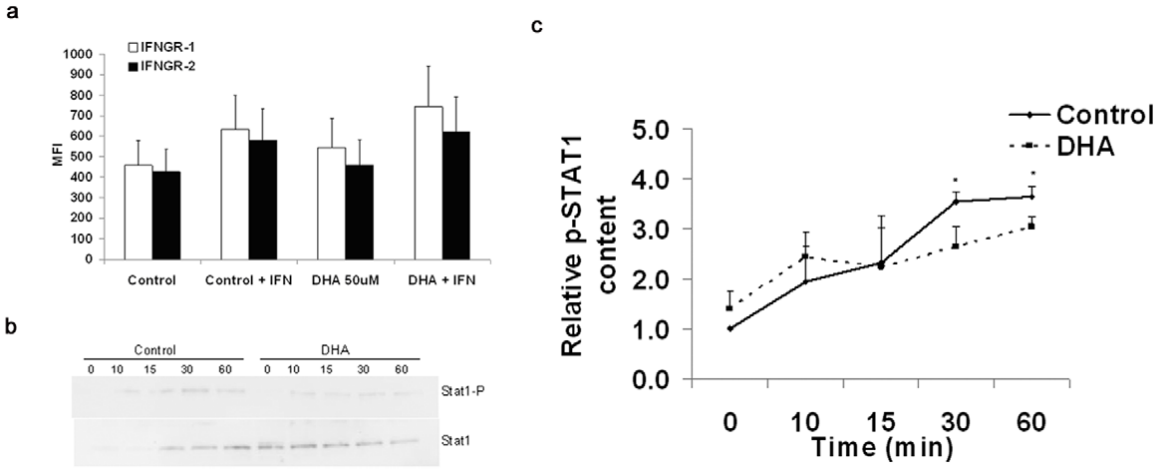
DHA does not affect the expression of IFNγR1 & R2 but decreases STAT1 phosphorylation in non-infected J774A.1 cells primed with IFNγ. Macrophages were incubated with 50uM DHA for 24 hours and then stimulated with or without IFNγ (100 U/ml) for 24 h. The cells were then labeled with PE-conjugated antibodies to IFNγR1 and IFNγR2 and intensity of fluorescence was quantified by flow cytometry, as described in the *Materials and Methods*. Cells were seeded at a density of 500,000 cells/well. a. Quantitative data represent the Mean Fluorescence Intensity (MFI) of PE fluorescence (mean ±SEM; n = 9) for IFNγR1 and IFNγR2. No significant differences were found between DHA and control groups. To measure STAT1 phosphorylation, cells were incubated with 50uM DHA for 24 hours and then stimulated with IFNγ (100 U/ml) from 0–60 min. The cells were then lysed in lysis buffer, loaded onto an SDS-PAGE gel, and transferred to a PVDF membrane as described in the *Materials and Methods*. b. Representative immunoblots probed for anti-phospho STAT1 (p-STAT1) or total STAT1 proteins. c. Quantitative data of the relative expression of p-STAT1 to total STAT1 (mean ±SEM; n = 6–9). Significant differences were found between control and DHA groups at 30 min and 1 h. Data are representative of three independent experiments. Abbreviations used: DHA, docosahexaenoic acid.

To better understand the underlying mechanisms by which DHA negatively affects IFNγ-mediated macrophage activation, non-infected J774.1 cells treated with DHA and activated with IFNγ were fluorescently stained with antibodies to IFNγR1 and IFNγR2. As shown in **Figure 6a**, DHA treatment alone did not affect the cell surface expression levels of IFNγR1 or IFNγR2 as assessed by mean fluorescent intensity. Subsequent activation with IFNγ slightly enhanced the surface expression of IFNγR1, but not IFNγR2, although this was not statistically significant. Therefore, the decrease in cytokine expression (**Fig. 1**) and increase in bacterial loads (**Fig. 3**) in DHA-treated, IFNγ-activated cells cannot be explained by the change in IFNγ receptor expression levels.

To determine whether IFNγ-induced cell signaling was affected by DHA treatment, non-infected cells were treated with DHA and activated with IFNγ at various time points. The lysates were immunoblotted with specific antibodies to total and phospho-STAT1 to determine the activation status of these cells. **Figure 6b** shows a representative image of the phospho- and total STAT1 content in J774.1 cells at intervals up to 60 min following IFNγ activation. DHA treatment had no affect on total STAT1 protein expression, and the expression of phosphorylated STAT1 peaked at 60 min post-activation in both DHA-treated and untreated cells. However, quantitative densitometry analysis (**Fig 6c**) indicated that DHA treatment significantly suppressed the phosphorylation status of STAT1 protein at both 30 min and 1 h post-activation with IFNγ. These data imply that DHA may impair downstream cell signaling events of the IFNγ receptor and, thus, suppress cell function.

### DHA interrupts colocalization of lipid rafts and IFNγ receptor

**Figure 7:**
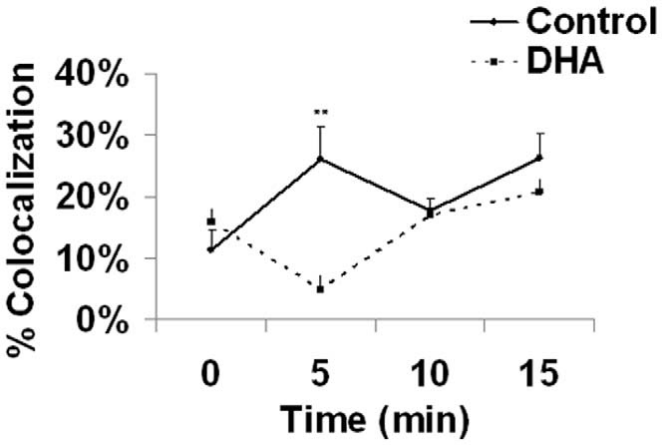
Colocalization of raft marker GM1 with IFNγR is impaired in DHA-treated, non-infected J774A.1 cells primed with IFNγ. Macrophages were incubated with 50uM DHA for 24 hours and then stimulated with IFNγ (100 U/ml) for0–15 min on chambered slides. The cells were then fixed, washed and immunostained with FITC-CTx (GM1) and IFNgR1 antibodies as described in the *Materials and Methods*. Images were then collected by confocal analysis at ×126 magnification. Colocalization of IFNγR1 with the raft marker was quantified as percentage of colocalized area (both green and red) over total raft area (green). Images were randomly captured from a minimum of 10 images per slide, with a minimum of 4 cells per image. Data are representative of three independent experiments. Significant differences were found between control and DHA groups at 5 min. Abbreviations used: DHA, docosahexaenoic acid.

The observation that DHA treatment did not affect total IFNγR expression, but did impair downstream signaling via STAT1, suggested that IFNγR function might be altered by DHA. To determine whether DHA affected recruitment of the IFNγR to lipid rafts during cell activation, DHA-treated, non-infected macrophages were activated with IFNγ for 0, 5, 10, and 15 min and fluorescently stained with anti-IFNγR1 antibody and anti-GM1, a lipid raft marker. Confocal analysis showed that the association of the IFNγ receptor with the lipid raft marker peaked at 5 min post-activation and steadily declined thereafter, demonstrating the rapid occurrence of these events at the cell membrane. The presence of DHA dramatically interrupted the interaction between IFNγ receptor and the lipid raft marker by ∼80% at 5 min post-activation (**Fig. 7**). At all other time intervals, colocalization events were similar in both the DHA and control group. Taken together, these data demonstrate that while DHA does not directly affect the surface expression levels of the IFNγ receptor, it does impair cell activation by disrupting the localization of the receptor to lipid rafts and thus, phosphorylation of key signaling molecules critical for cell function.

## Discussion

Malnutrition [2], hypercholesterolemia [3], and diabetes [4] are examples of host nutritional conditions associated with increased susceptibility to TB. Although dietary n-3 PUFA are frequently used as anti-inflammatory and tumor-suppressive treatments [7], [32], their immunosuppressive effects might negatively impact the host defense against *Mtb*, in part, by interfering with activation of macrophages. Our results support this conclusion. We demonstrated that IFNγ-activated macrophage-like J774A.1 cells treated with DHA exhibited impairment in several indicators of cell activation and increased susceptibility to virulent *Mtb* infection. DHA treatment was associated with down-regulated pro-inflammatory cytokine production, reduced antimicrobial capacity, a weak oxidative response, diminished phagolysosome maturation, and interrupted signaling events critical for IFNγ-induced cell activation. The immunosuppressive effect was unique to DHA, per se, and not fatty acids in general. J774A.1 cells treated with arachidonic acid (AA; 20:4 n-6) or palmitic acid (PA; 16:0) responded essentially the same as untreated cells and significantly different from DHA-treated cells, as shown in Supplemental Figures S1, S2, and 3. Therefore, the suppressive effects on IFNγ-induced macrophage activation reported here are unique to DHA. This is in agreement with previous studies done by our group in which other fatty acids were used as controls to demonstrate a DHA-specific effect on T-cell activation [33], [34].

Our data are consistent with previous reports in which dietary n-3 PUFA compromised host resistance to intracellular infections. Mice fed with n-3 PUFA exhibited increased susceptibility to *Listeria monocytogenes* [17], *Herpes simplex type I* [35], *Salmonella typhymurium* [16], *Paracoccidioides brasiliensis* [36] and reovirus [37]. In agreement with our findings, these studies also reported reduced production of pro-inflammatory cytokines, low expression of accessory molecules and defective cell activation following n-3 PUFA feeding. Indeed, an increased dietary intake of n-3 PUFA impaired resistance to TB in guinea pigs [12], [38], [39] and mice [11] infected with virulent *Mtb*.

Cytokines such as TNFα [40] are not only markers of activation, but also are essential to induce an effective macrophage killing activity and for the development of a protective response against TB. Since TNFα production was impaired in our studies (**Fig. 1**), it is reasonable to propose that the bacterial control could be also affected by DHA through this mechanism. Similar changes in TNFα levels were recently reported in *Mtb*-infected J774A.1 cells treated with another n-3 PUFA, eicosapentaenoic acid (EPA) [6]. The levels of incorporated DHA achieved in our study are similar to previous reports in both murine [30], [31], guinea pig [12] and human cells [41], which reported DHA levels exceeding 10 mol% *in vivo* following fish oil feeding. In addition, our experimental conditions (BSA-complex system and 24 h incubation) have been shown to maximize fatty acid incorporation [42], [43] and the J774A.1 cell line is a well-established model for studies of mycobacterial pathogenesis and immune response [44], [45], as well as lipid metabolism [5], [43]. However, experiments beyond the scope of the present study will be necessary to determine the relevance of our findings in primary lung or alveolar macrophages. The finding of an increased bacterial internalization and permissiveness in DHA-treated macrophages is supported by previous studies in which DHA increased phagocytic activity [30], [46]. This finding raises the question of which specific event during bacterial internalization [47] is being modulated by DHA; e.g. binding to the cell surface, membrane invagination, phagosome formation, phagolysosomal fusion, etc [47]. Based on our data, it is unclear which event or events are modulated and further experiments are necessary. The CFU results (**Fig. 3C**) are consistent with the loss of mycobacterial control in DHA-cultured cells, reported by Anes et al [5]. Jordao et al. reported reduced antimycobacterial activity in J774A.1 cells treated with the n-3 PUFA, EPA [6]. Although we used very different experimental conditions, our results are complementary to those of Anes et al. and add significantly to the understanding of the n-3 PUFA effects.

The distribution of acidotropic probes and the recruitment of endosomal proteins to mycobacterial phagosomes during the maturation process have been well described. Lysotracker is an acidotropic probe which accumulates into acidic compartments. Lamps are membrane glycoproteins that accumulate into lysosomes and late endosomes [48] and Rabs are small GTP binding proteins which play a role in regulating membrane fusion events within endocytic compartments [49]. Lysotracker is commonly used as an indicator of phagosome maturation and acidification in IFNγ-activated cells. Although its accumulation overlays compartments positive for specific lysosomal markers [15], [49], the lysotracker lacks specificity and needs to be validated by detecting lysosomal proteins. We confirmed that robust markers of phagosomal maturation like Rab7, LAMP1 and LAMP-3 were excluded from mycobacterial phagosomes in DHA-treated cells, showing reduced fusion of *Mtb*-containing phagosomes with late endosomal compartments. However, since only one time interval was evaluated, we cannot rule out transient or delayed recruitment of those markers under DHA treatment.

Diverse molecular mechanisms at different cellular levels might explain the observed DHA effect [50]. n-3 PUFA have been shown to promote apoptosis in T cells [29] and colonocytes [51] and to reduce cell attachment and proliferation. It is important to note that we found no significant differences in cell viability, proliferation or adherence among treatment groups (Data not shown). It has been shown that DHA can modulate membrane fluidity, permeability, and the distribution and activation of transmembrane proteins, receptors and channels [52] and different membrane-associated processes, [53],[54]. We have found that DHA did not mediate any changes in IFNγ receptor surface expression (**Fig. 6**) in non-infected cells. Similar findings were reported in the peritoneal macrophages and splenocytes of n-3 PUFA-fed IFNγ knockout mice [55]. DHA did, however, downmodulate the IFNγ-mediated STAT1 signaling pathway by decreasing STAT1 phosphorylation (**Fig. 7**), a critical pathway induced by IFNγ receptor activation. Accordingly, n-3 PUFA reduced STAT-1 phosphorylation in the peritoneal macrophages of n-3 PUFA-fed IFNγ knockout mice [56]. This may be explained, in part, by the disrupted interaction between the IFNγ receptor and lipid rafts (**Fig. 7**). Upon cell activation, lipid rafts compartmentalize critical signal transducing molecules to form the immunological synapse. We have recently reported that dietary n-3 PUFA modulate T-cell lipid rafts and suppress the localization and activation of signaling proteins such as PKCθ and PLCγ1 [33], [54]. These molecular mechanisms may also be involved in the decreased STAT1 phosphorylation and subsequent downstream activation events in DHA-treated macrophages and remain to be elucidated.

Current interest in n-3 PUFA focuses on their protective properties against a wide range of inflammatory and autoimmune diseases. Indeed, the U.S. Food and Drug Administration (FDA) permits the inclusion of information regarding omega-3 health benefits on labels for foods supplemented with n-3 PUFA. Moreover, Lovaza®, a drug which contains DHA and EPA, has been approved for use in humans to reduce very high triglycerides levels. However, our findings raise questions regarding the safety of n-3 supplementation. An improved understanding of the lipid effect on intracellular infections, including TB, will allow a better assessment of the potential health risks of excessive intake of n-3 PUFA in humans, leading to the establishment of dietary guidelines in order to avoid detrimental effects. We hypothesize that diets supplemented with n-3 PUFA increase host susceptibility to TB infection *in vivo*. However, our *in vitro* findings reflect just partially the complexity of the immune response in multicelullar hosts and need to be validated using *in vivo* systems. Further investigation will be required to establish the effect of n-3 PUFA on resistance to TB in relevant animal models and to determine what impact, if any, n-3 PUFA supplementation has in people at risk of infection.

## Materials and Methods

### Cells

The murine-macrophage-like cell line J774.A1 (ATCC TIB-67) was maintained in RPMI (Invitrogen) supplemented with 10% heat-inactivated fetal bovine serum (FBS) (Atlanta Biol.) and 1% penicillin-streptomycin (Gibco), in 5% CO_2_ at 37°C. Cells were seeded in plates or glass coverslips and allowed to adhere overnight. Cell monolayers were incubated for 24 h with 50 uM conjugated BSA-DHA (docosahexaenoic acid; 22:6n-3) [21], [57], BSA-AA (arachidonic acid; 20:4n-6), BSA-PA (palmitic acid; 16:0) or BSA alone. Fatty acids (FA) were conjugated at a FA∶BSA 3∶1 mole ratio and resuspended in RPMI, to mimic physiological conditions according to Arrington et al [21], [42]. The fatty acid concentration used mimics the levels reached in human cells supplemented with DHA [21], [41], [57] and the 24 h incubation time was selected to maximize the incorporation of fatty acids based on previous reports [43]. The fatty acid-treated cells were then incubated with or without IFNγ (100U/ml Biosource) to induce activation. AA, and n-6 PUFA, was included to control for a general polyunsaturated fatty acid effect. PA, a saturated fatty acid, was used to control for a general unsaturated fatty acid effect.

### Fatty acid profiles

Total lipids were extracted from fatty acid-treated J774A.1 cells by the method of Folch [58]. Phospholipids were separated by TLC, using chloroform/methanol/acetic acid/water as solvents. Transesterification of the isolated phospholipids was performed in presence of 6% methanolic HCl. Fatty acid methyl esters were analyzed by capillary gas chromatography as previously described [4], [59]. Peaks of resolved fatty acids were identified by comparison with standards (Nu Chek Prep).

### Infection

The virulent strain H37Rv of *M. tuberculosis* (ATCC 27294) was cultured in Middlebrook 7H9 medium (Becton Dickinson) and stocks were prepared and stored at −80°C before use, according to Grover et al [60]. For infection, bacteria were thawed, vortexed, sonicated and passed through a 28G needle fifteen times to disrupt bacterial clumps, as previously described [61]. Macrophages were infected at a multiplicity of infection (MOI) of 10 or 20, according to the type of experiment. Before infection, non-incorporated fatty acids were removed from the culture and infected cells were maintained in RPMI (Gibco) supplemented with 2% heat-inactivated FBS (Atlanta Biol). At specific time points after infection, cell monolayers were washed with PBS (Gibco) and incubated with 50 µg/ml gentamicin (Gibco) to kill extracellular bacteria [62].

### Cytokine production

Culture supernatants were harvested and assessed for IL-6, IL-12p70, TNFα, MCP-1, IL-10 and IFNγ by flow cytometry using the Cytometric Bead Array CBA mouse inflammation kit (Becton Dickinson) according to the manufacturer's protocol. The cytokine concentration was determined by using the CBA software based on calibration curves and the four-parameter curve fit option. Supernatants from unstimulated cells were used to establish basal levels of cytokine expression.

### Costimulatory molecule and IFNγ receptor expression

Monolayers were scraped and cells were resuspended in PBS. Cells were incubated with phycoerythrin (PE)-conjugated rat anti-mouse antibodies to CD14, CD40, CD86 (Becton Dickinson) and IFNγRα and IFNαRβ (Santa Cruz Biotechnology). Cells were pretreated with a rat anti-mouse CD16/CD32 to block non-specific binding and a PE-conjugated rat IgG (Becton Dickinson) with low background binding used as isotype control. Cells were fixed in 2% paraformaldehyde in PBS and analyzed using a FACSCalibur flow cytometer (Becton Dickinson). CellQuest software was used to determine both intensity and percentage of PE fluorescence.

### Bacterial permissiveness

Cells were seeded in Petri dishes (Fisher) or on glass coverslips (Corning) at a density of 5×10^5^ cells and left to form monolayers overnight. Monolayers were infected with a green fluorescent protein (GFP)-expressing virulent strain (H37Rv) of *Mycobacterium tuberculosis*, kindly provided by Dr. Scott Franzblau (University of Chicago) [63]. Nuclei were counterstained with 1 mM Hoescht 33342 (Fluka). Discrimination between intracellular and extracellular bacteria was accomplished by using simultaneously a polyclonal primary antibody E193.1.11.WCL against whole cell lysate of *Mtb*, kindly provided by Dr Karen Dobos-Elder (Colorado State University). Serial sections across entire cells were taken with a Z-stack spanning a thickness of 15 um and a step size of 0.5 um, and 3D reconstruction was performed to confirm the intracellular bacterial localization. The percentage of cells with internalized bacteria was determined in an Olympus disk scanning confocal microscope by using Slidebook software and by flow cytometry using CellQuest software.

### Mycobacterial survival

After infection, monolayers were lysed with 0.1% SDS (Sigma). Lysates were serially diluted and plated on 7H10 Middlebrook agar (Becton Dickinson). The colony forming unit (CFU) counts were determined after 3–4 weeks of incubation at 37°C in 5% CO_2_, as described previously [40]. Data are expressed as mean log_10_ viable CFU.

### Phagolysosome maturation

Cells were infected with GFP-expressing bacteria and incubated with 50 nM Lysotracker Red DND99 (Invitrogen) for 2 h before and during the infection. Alternatively, cells were fixed with 3.7% formaldehyde, permeabilized with 0.2% Triton X-100 (Sigma) and blocked with 2% BSA fraction V (Sigma) and 0.02% Tween-20 in MgPBS before incubation with primary antibodies against LAMP-1 (Sigma), Rab5, Rab7 and LAMP-3 (SantaCruz Biot), as described by Vergne et al [14]. A secondary PE-conjugated antibody (Becton Dickinson) was added and nuclei were counterstained with 1 mM Hoescht 33342 (Fluka). Coverslips were mounted onto slides using Slowfade Gold mounting medium (Invitrogen) and analyzed on an Olympus disk scanning confocal microscope by using Slidebook software. The quantitative scoring of colocalization was done by determining the percentage of lysosomal marker- positive mycobacterial phagosomes. For each group, at least 100 *Mtb*-containing phagosomes were counted and scored for the presence of lysosomal markers. Serial sections were taken with Z-stack spanning to confirm colocalization.

### Reactive oxygen species (ROS) production

Cells were infected and intracellular ROS levels were measured by staining with oxidative fluorescent dyes 10 uM 5-(and 6-)-chloromethyl-2′,7′-dichlorodihydrofluorescein diacetate acetyl ester (DCFDA) (Invitrogen) and 5uM Dihydroethidium (DHE) (Invitrogen), according to Carter et al [64]. DHE becomes a fluorescent compound (Ethidium bromide, EB) after oxidation by reactive species, including O^2−^, H_2_O_2_ and NO. The intensity of red EB fluorescence is proportional to ROS concentration. DHE oxidation can occur due to basal macrophage metabolic activity [65], therefore background was subtracted using uninfected cells as control. Cells were fixed in 2% paraformaldehyde in PBS and analyzed using a FACSCalibur flow cytometer (Becton Dickinson). CellQuest software was used to determine both intensity and percentage of EB fluorescence.

### Western blot analysis

Monolayers of J774.1 macrophages treated with IFNγ for various intervals (0, 10, 15, 30 and 60 min) were resuspended in RIPA lysis buffer (Santa Cruz Biotechnology) and cell lysates stored at −80°C until further analysis. Cell lysates were electrophoresed on a 4–12% Tris-Glycine gel (Invitrogen) and transferred to a PVDF membrane for immunoblotting. The membranes were incubated overnight in a 1∶500 dilution of anti-phospho-STAT1 or total STAT1 antibody (Millipore/Upstate) followed by a 1 h incubation with goat anti-rabbit 2° antibody conjugated to HRP (1∶1000). Chemiluminescence substrate (Super Signal West Femto; Pierce) was used to develop the reaction and bands were captured on a BioRad Imager and quantified using QuantityOne software (BioRad laboratories).

### Colocalization experiments

J774.1 macrophages were seeded onto poly-L-lysine coated cover glass slides as described previously [54]. After cells were allowed to adhere for 30 min, they were treated with DHA and IFNγ as described previously. Cells were then fixed in 4% paraformaldehyde for 20 min, rinsed with PBS, and incubated with 10 nmol/L glycine in PBS for 10 min to quench aldehyde groups. Cell membranes were permeablized by exposure to 0.2% Triton X-100 in PBS for 5 min at RT, followed by PBS washing. Cells were subsequently covered with blocking solution (1% BSA/0.1% NaN_3_ in PBS) and incubated at 4°C overnight with anti-mouse IFNγR antibody (Invitrogen). After PBS washing, cells were incubated with secondary AlexaFluor 568 goat Ab to rabbit IgG (Molecular Probes). To visualize ganglioside (GM1) localization, cells were incubated with GM1-specific cholera toxin (CTx) B subunit-FITC (CTx-FITC; Sigma-Aldrich), respectively. Following serial ethanol dehydration steps, samples were mounted onto glass slides with ProLong Antifade reagent (Molecular Probes). Slides were stored at −20°C until analysis by confocal microscopy. Fluorescence images were acquired at ×126 magnification on a Bio-Rad Radiance 2000 MP confocal microscope as previously described [33]. For FITC detection, an excitation wavelength was set to 488 nm and emission was collected with a 550LP dichroic mirror and 515/28-nm BP filter. For Alexa 568 detection, an excitation of 568 nm was used, and emission was collected using a 600 nm LP filter. Ten images, with a minimum of four cells per image, were collected randomly per treatment group. Data are representative of 3 independent experiments. For data analysis, regions of interest were selected by drawing polygons around each cell boundary. Colocalization of IFNγ with the raft marker in each cell was quantified as percentage of colocalized pixel area (both green and red) over total raft pixel area (green) according to the algorithm provided with the Meridian Ultima Work Station (Meridian Instruments).

### Statistical Analysis

Data were statistically evaluated with Excel or GraphPad Prizm software, version 4.0. Comparisons between more than two groups over time were assessed by ANOVA and Tukey-Kramer test was performed when ANOVA results were significant. Differences between treatments and control were analyzed by using the Student's t-test for samples with equal variance, considering *p* values <0.05 as significant. Data are presented as means and error bars represent standard errors of three independent experiments.

## Supporting Information

Figure S1 Suppression of TNFα production and CD40 and CD86 expression by DHA in infected J774A.1 cells after IFNγ treatment. Macrophages were incubated with 50uM DHA, AA or PA for 24 h and infected with M. tuberculosis for 1 h. Culture supernatants were quantified for TNFα or incubated with PE-conjugated antibodies to CD40 and CD86 and fluorescence intensity was quantified by FACS analysis, as described in the Materials and Methods. a. Quantitative data represent the concentration of TNFα post infection (pictograms per milliliter; mean±SEM; n = 9).* Indicates a significant effect of DHA within the same interval compared to Control, PA, or AA groups ** p<0.05. b. Quantitative data represent the relative percentage of PE fluorescence (mean±SEM; n = 9) for CD40 and CD86. * Indicates a significant effect of DHA compared to Control, PA and AA groups * p<0.01** p<0.05. Data are representative of three independent experiments. Abbreviations used: PA, palmitic acid; AA, arachidonic acid; DHA, docosahexaenoic acid; PE, phycoerythrin.
(1.66 MB TIF)

Figure S2 DHA reduces mycobacterial killing and impairs phagolysosmal fusion in IFNγ-stimulated J774A.1 macrophages. Cells were incubated with 50uM DHA, AA or PA for 24 h and infected with 10 MOI GFP-expressing M. tuberculosis for 1 h. a. The relative percentage of Mtb-infected cells was quantified by flow cytometry, as described in the Materials and Methods. Quantitative data represent the percentage of infected cells (mean±SEM; n = 9). b. Phagolysosome maturation was defined based on acquisition of lysotracker by GFP-Mtb-containing phagosomes and visualized by fluorescent microscopy, as described in the Materials and Methods. Percentages of Lysotracker-, LAMP-1, LAMP3-, Rab5-, Rab7-positive mycobacterial phagosomes. Data represent the percentage of colocalization (mean±SEM; n = 5). ).* Indicates a significant effect of DHA within the same interval compared to Control, PA, or AA groups * p<0.01, ** p<0.05, ***P<0.001. Data are representative of three independent experiments. Abbreviations used: PA, palmitic acid; AA, arachidonic acid; DHA, docosahexaenoic acid.
(4.85 MB TIF)

Figure S3 DHA impairs oxidative metabolism in infected J774A.1 cells activated with IFNγ. Cells were incubated with 50uM DHA, AA or PA for 24 h and infected with 20 MOI Mtb. Reactive oxygen intermediates were estimated by FACS, as described in the Materials and Methods. Quantitative data show the relative percentage of EB red fluorescence (mean±SEM; n = 9). * Indicates a significant effect of DHA compared to the other three treatment groups, *p<0.01. Data are representative of three independent experiments. Abbreviations used: PA, palmitic acid; AA, arachidonic acid; DHA, docosahexaenoic acid.
(0.48 MB TIF)
