# Supplementary figures and images for: Incorporation of a Dietary Omega 3 Fatty Acid Impairs Murine Macrophage Responses to Mycobacterium tuberculosis
Source: PLoS One. 2010 May 28;5(5):e10878. doi: 10.1371/journal.pone.0010878 (PMC2878322; doi:10.1371/journal.pone.0010878)

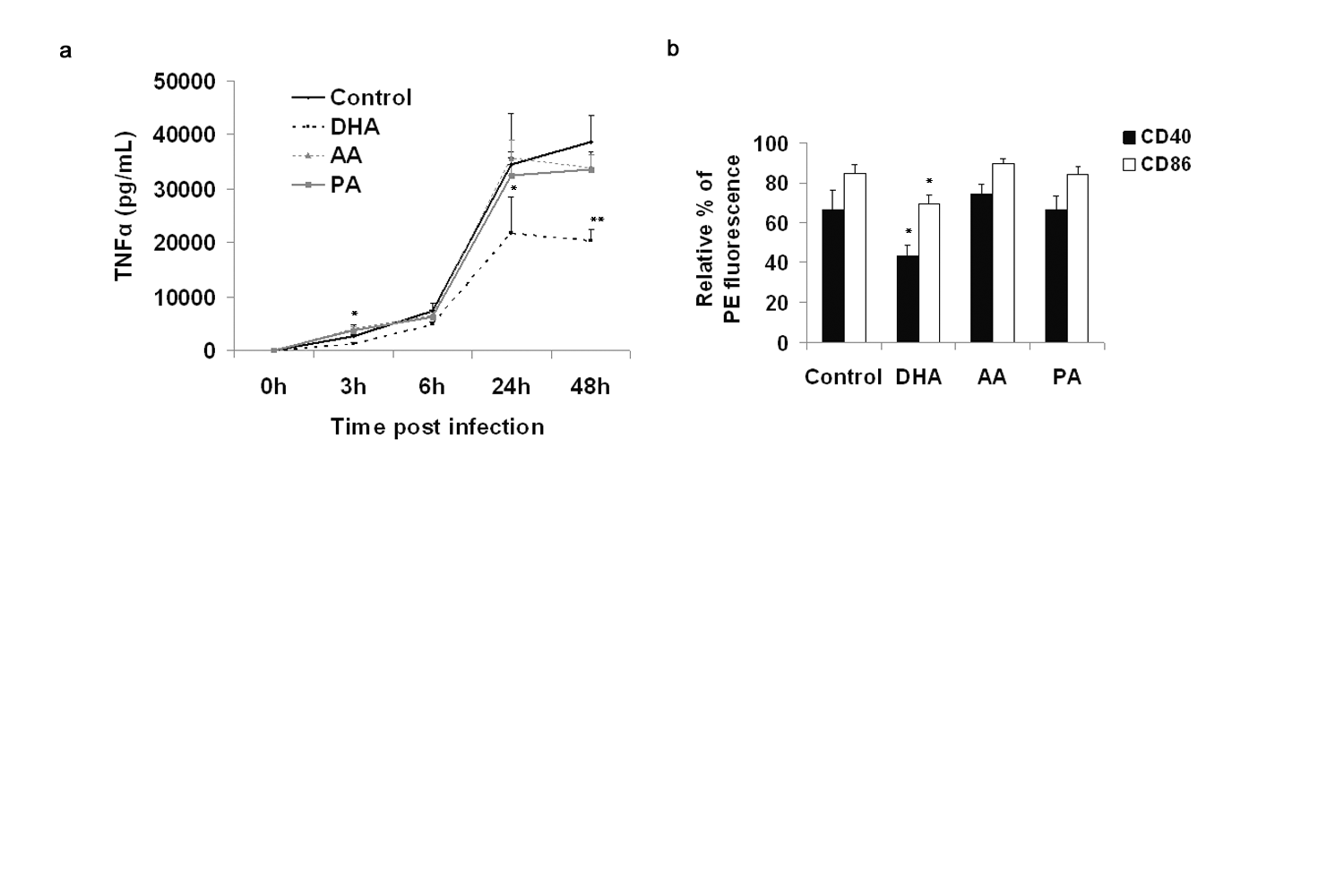

Supplement: Figure S1 — Suppression of TNFα production and CD40 and CD86 expression by DHA in infected J774A.1 cells after IFNγ treatment. Macrophages were incubated with 50uM DHA, AA or PA for 24 h and infected with M. tuberculosis for 1 h. Culture supernatants were quantified for TNFα or incubated with PE-conjugated antibodies to CD40 and CD86 and fluorescence intensity was quantified by FACS analysis, as described in the Materials and Methods. a. Quantitative data represent the concentration of TNFα post infection (pictograms per milliliter; mean±SEM; n = 9).* Indicates a significant effect of DHA within the same interval compared to Control, PA, or AA groups ** p<0.05. b. Quantitative data represent the relative percentage of PE fluorescence (mean±SEM; n = 9) for CD40 and CD86. * Indicates a significant effect of DHA compared to Control, PA and AA groups * p<0.01** p<0.05. Data are representative of three independent experiments. Abbreviations used: PA, palmitic acid; AA, arachidonic acid; DHA, docosahexaenoic acid; PE, phycoerythrin. (1.66 MB TIF) [file pone.0010878.s001.tif]

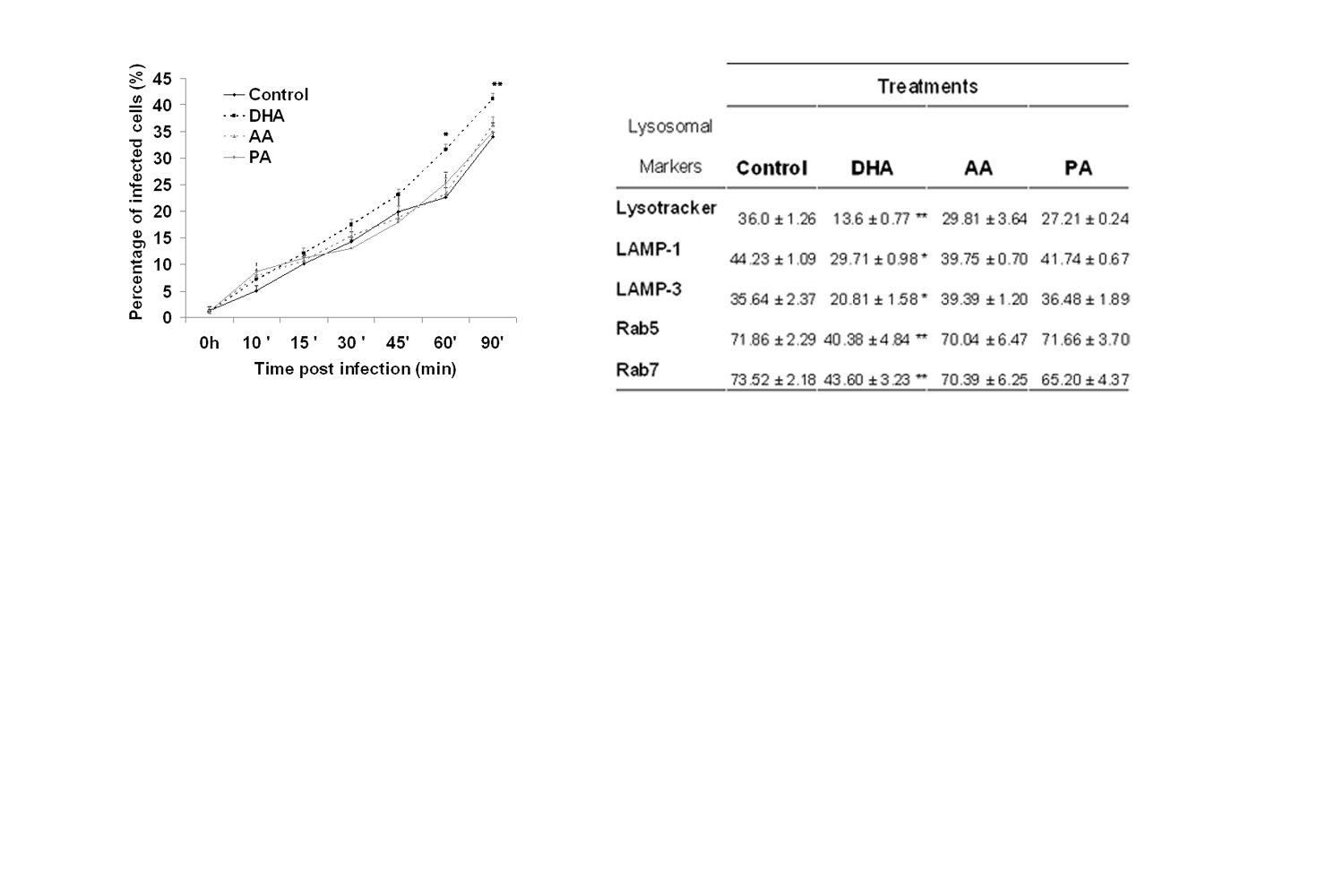

Supplement: Figure S2 — DHA reduces mycobacterial killing and impairs phagolysosmal fusion in IFNγ-stimulated J774A.1 macrophages. Cells were incubated with 50uM DHA, AA or PA for 24 h and infected with 10 MOI GFP-expressing M. tuberculosis for 1 h. a. The relative percentage of Mtb-infected cells was quantified by flow cytometry, as described in the Materials and Methods. Quantitative data represent the percentage of infected cells (mean±SEM; n = 9). b. Phagolysosome maturation was defined based on acquisition of lysotracker by GFP-Mtb-containing phagosomes and visualized by fluorescent microscopy, as described in the Materials and Methods. Percentages of Lysotracker-, LAMP-1, LAMP3-, Rab5-, Rab7-positive mycobacterial phagosomes. Data represent the percentage of colocalization (mean±SEM; n = 5). ).* Indicates a significant effect of DHA within the same interval compared to Control, PA, or AA groups * p<0.01, ** p<0.05, ***P<0.001. Data are representative of three independent experiments. Abbreviations used: PA, palmitic acid; AA, arachidonic acid; DHA, docosahexaenoic acid. (4.85 MB TIF) [file pone.0010878.s002.tif]

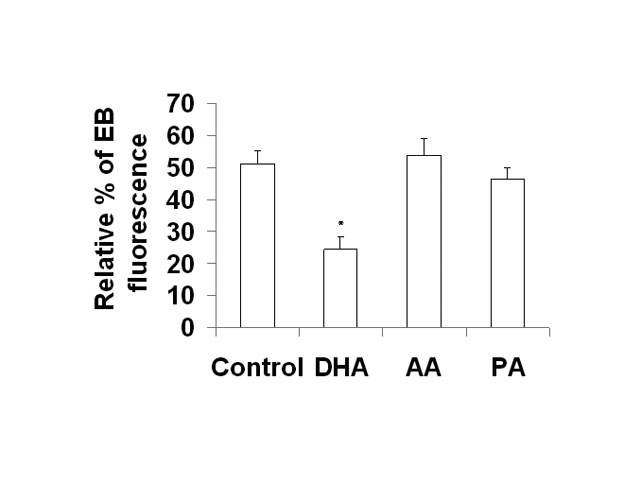

Supplement: Figure S3 — DHA impairs oxidative metabolism in infected J774A.1 cells activated with IFNγ. Cells were incubated with 50uM DHA, AA or PA for 24 h and infected with 20 MOI Mtb. Reactive oxygen intermediates were estimated by FACS, as described in the Materials and Methods. Quantitative data show the relative percentage of EB red fluorescence (mean±SEM; n = 9). * Indicates a significant effect of DHA compared to the other three treatment groups, *p<0.01. Data are representative of three independent experiments. Abbreviations used: PA, palmitic acid; AA, arachidonic acid; DHA, docosahexaenoic acid. (0.48 MB TIF) [file pone.0010878.s003.tif]
